# Laparoscopic incisional and ventral hernia repair

**DOI:** 10.4103/0972-9941.37190

**Published:** 2007

**Authors:** Amrit Pal Singh Bedi, Tahir Bhatti, Alla Amin, Jamal Zuberi

**Affiliations:** Diploma Laparoscopic Surgery, France; * Department of General Surgery, Hemel Hempstead NHS, Hillfield Road, HP2 4AD UK

**Keywords:** Incisional hernia, laparoscopy, mesh, repair, ventral hernia

## Abstract

**Background:**

It has been more than a decade, since the introduction of laparoscopic management of ventral and incisional hernia. The purpose of this article was to systematically review the literature, analyze the results of Laparoscopic repair of ventral and incisional hernia and to ascertain its role.

**Materials and Methods:**

Pubmed was used for identifying the original articles. Both incisional and ventral hernia repair were included. Out of 145 articles extracted from Pubmed, 34 original studies were considered for review. More than three thousand patients were included in the review. Variables analyzed in the review were inpatient stay, defect size, mesh size, hematoma, seroma, wound infection, bowel perforation, obstruction, ileus, recurrence and pain. Qualitative analysis of the variables was carried out.

**Results:**

Seromas (5.45%) and post operative pain (2.75%) are the two common complications associated with this procedure. Recurrence rate was found to be 3.67%. Overall complication rate was 19.24%, with two deaths reported.

**Conclusion:**

The results suggest laparoscopic repair of ventral and incisional hernia as an effective procedure. Faster recovery and shorter in patient stay - makes it a feasible alternative to open repair.

To review the literature from 1995 to 2006 in a systematic manner and to analyze the results of laparoscopic incisional and ventral hernia repair.

## INTRODUCTION

Postoperative incisional hernia is one of the most common surgical procedure being performed in General Surgery. The incidence of incisional hernia, as reported in literature is 3% to 20%.[1–9] The principle of laparoscopic incisional hernia repair is based on Rives-Stoppa repair, first published in 1985.[10] Original Rives-Stoppa repair involved extensive tissue dissection in a myofascial plane for placement of mesh. LeBlanc and Booth[5] first described laparoscopic repair of incisional hernia in 1993. Since than, many authors have published reports of laparoscopic incisional and ventral hernia repair (LIVHR). This procedure is fast emerging as an alternative to open technique. The purpose of this study was to review and analyze the results of different authors and determine the clinical status of laparoscopic incisional and ventral hernia repair.

## MATERIALS AND METHODS

English Language articles on Laparoscopic Incisional or Ventral hernia repair (LIVHR) from 1995 to 2006 were extracted from electronic databases. Pubmed and MEDLINE search was done using following terms “incisional hernia”, “ventral hernia”, “laparoscopy” and “repair” to find out relevant laparoscopic studies. Original prospective and retrospective observational studies and randomised control trials were included. Comparison studies between laparoscopic and open incisional/ventral hernia repair were also included. Laparoscopic repair data was extracted from comparison studies. In total 145 articles were found of interest. A cross-reference search was also carried out from the latter to include relevant manuscripts. Three different senior surgeons assessed these articles.

Review of literature or meta-analysis (only one found) was not included to avoid repetition bias. Studies that were excluded were animal experimental models, articles containing combined abdominoplasty or autoplasty, case reports and primary repair using suture. The latter exclusions were done to make the review more specific and clinically-oriented. Articles with less than 10 reported cases were also excluded from the study, to avoid bias from authors being in the learning curve.

**Figure 1:**
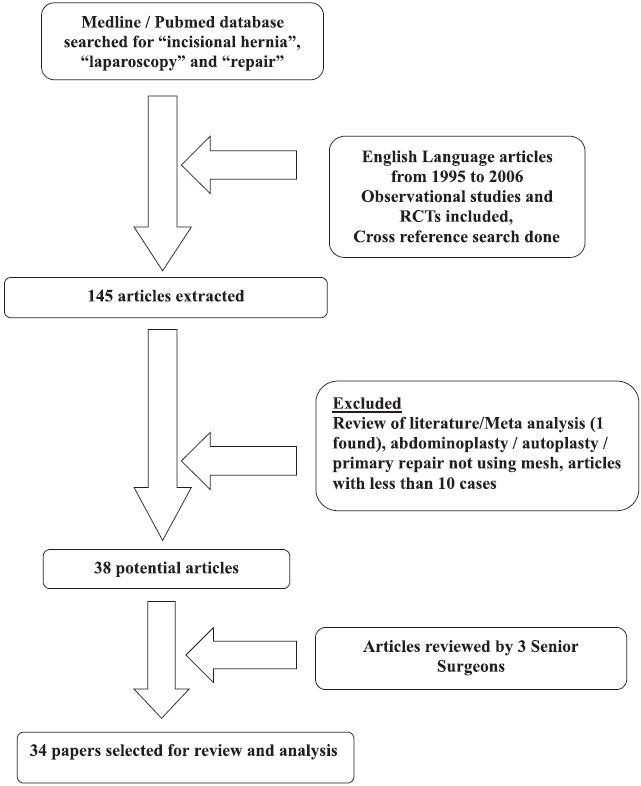
Quorom flow diagram

Thirty four articles were finally included into the study based on the above criteria (Quality of Reporting of Meta-Analysis, QUOROM, flow diagram [Figure 1]). The following parameters were studied including inpatient stay, defect size, mesh size, hematoma, seroma, wound infection, bowel perforation, ileus, obstruction, recurrence, chronic pain and other complications. Though quantitative analysis was not performed, qualitative assessment of parameters was done.

## RESULTS

**Figure 2:**
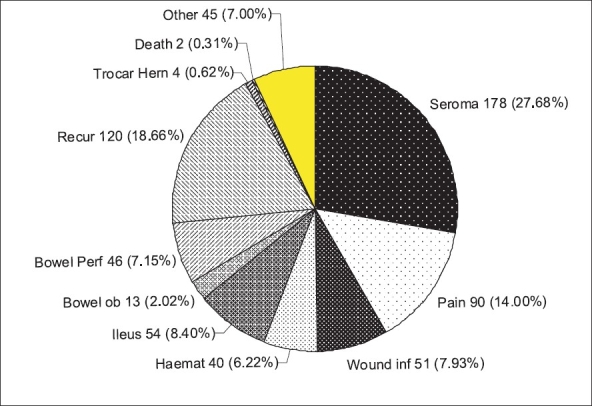
Pie chart showing percentage distribution of 643 complications

3266 patients were analyzsed from 34 studies with an average age of 55.34 years. One hundred and eighty extra procedures were performed while doing the primary operation. Further results are summarised in Tables 1–5 and [Figure 2].

**Table 1:** Studies with more than 25 cases

Author	(Reference) year	Study type	Patients number	Follow up in (months)
Moreno Egea *et al.*[11]	2002	*prospective	55	18
Aura *et al.*[12]	2002	*prospective	86	37
B. Kuae *et al.*[13]	2001	*retrospective	30	12
Bageacu *et al.*[14]	2002	*retrospective	159	49
Bamehriz *et al.*[15]	2004	*prospective	28	10.7
Ben-Haim *et al.*[16]	2002	*retrospective	100	19
Bower *et al.*[17]	2004	*retrospective	100	6.5
Chowbey *et al.*[18]	2003	*retrospective	34	median 16.5
Eid *et al.*[19]	2003	*retrospective	79	34
Franklin *et al.*[20]	2004	*retrospective	384	47.1
Franklin *et al.*[21]	1998	*retrospective	176	NR#
Heniford *et al.*[22]	2000	*retrospective	415	23
Koehler *et al.*[23]	1999	*retrospective	32	20
Le Blanc *et al.*[24]	2003	*retrospective	100	36
Le Blanc *et al.*[25]	2000	*retrospective	100	51
Mc Greevy *et al.*[26]	2003	**comparison	65	1
Mizrahi *et al.*[27]	2003	*retrospective	231	NR#
Muysoms *et al.*[28]	2004	*retrospective	50	14.3
Park *et al.*[29]	1998	**comparison	56	24.1
Park *et al.*[30]	1996	*retrospective	30	8
Parker *et al.*[31]	2002	*retrospective	50	41
Raftopoulos *et al.*[32]	2002	*retrospective	50	20.8
Riet *et al.*[33]	2002	**comparison	25	16
Olmi *et al.*[34]	2005	**comparison	50	median 9
Verboe *et al.*[35]	2004	*prospective	45	NR#
Misra *et al.*[41]	2006	*prospective	33	13.7
Lomanto *et al.*[42]	2006	*prospective	50	19.6
Earle *et al.*[43]	2006	*retrospective	469	NR#
Motson *et al.*[44]	2006	*prospective	117	median 42

* Observational,

** Comparison study between laparoscopic and open repair,

NR# not recorded in study

**Table 2:** Results

Results	Mean value	Comment
Defect size*	108. 74 square cm	Defect average area from 15 studies; defect diameter as 7.05 cm in 5 studies
Mesh size*	268.61 square cm	Area evaluated in 10 studies
Operating time*	96.52 minutes	Evaluated in 29 studies, could not be evaluated in 2 studies, mentioned as median in another 3
Hospital stay*	54.85 hrs	Evaluated in 26 studies**
Follow up*	29.7 months	Follow up of 26 studies**

* All of included 34 studies did not mention this parameter,

** Mentioned as median or range in other studies.

**Table 3:** Complications

Complication	Number(3266)#	Studies*	Percentage
Seroma	178	29	5.45
Postoperative pain	90	17	2.75
Wound infection and Trocar site cellulitis	44 + 7 = 51	25	1.56
Haematoma	40	13	1.2
Ileus	54	17	1.65
Bowel obstruction	13	9	0.39
Bowel Perforation	46	17	1.40
Recurrence	120	27	3.67
Trocar site hernia	4	3	0.12
Death	2	2	0.06
Other	45	12	1.37
Total	643		19.68

# Total number of cases in 34 studies,

* Studies reporting this complication

**Table 4:** Complications as noted in different studies

Author	Seroma	Postoppain	Wound infection	Trocar cellul	Ileus	Obstrn	Recur
Moreno-egea[11]	5				1		1
Aura[12]	12	5			1		6
B. kua[13]		1	2			1	3
Bageacu[14]	22	31	4		6	2	19
Bamehriz[15]	9	2					1
Ben-Haim[16]	11				4	3	2
Bower CE[17]	1	3	2		2	2	2
Chari[36]		0	1	0			
Chowbey[18]	7		1				1
Eid[19]	3	3			1		4
Franklin[20]	12	12	3		5	1	11
Franklin[21]	2		4				2
Heniford[22]	8	8	4	5	9		14
Holzman[37]	1	4	1			1	2
Koehler[23]	2			2		1	3
Lau[38]	2		1				
Le Blanc[24]	7	2	2		9	1	4
LeBlanc[25]	7		1		2		9
Mc Greevey[26]	2	0	2	0	0		0
Mizrahi[27]	10						8
Muysoms[28]	2	4	0	0	2		1
Tagaya[39]	1		1 (abscess)				2
Park[29]	2	2	2		3		6
Park[30]		1	1		3		1
Parker[31]	1	2					0
Raftopoulos[32]	7		2		3		1
Riet[33]	9		1		1		4
Stefano olmi[34]	6	1	1		0		1
Tsimoyiannis[40]	1		1				
Verbo[35]	3		0	0	1		0
Misra[41]	4	6	2				2
Lomanto[42]	5		2		1		1
Earle[43]							
Motson[44]	14	3	3			1	9
Total	178	90	44	7	54	13	120

**Table 5:** Type of mesh used in the 34 studies

Mesh type	Number of studies
ePTFE (expanded polytetrafluoroethylene)	15
Polypropylene	10
Dual mesh/composite	8
Goretex	4
Marlex	2

## DISCUSSION

### Technique

To gain intra abdominal access, peritoneal insufflation is done usually away from the hernia site.[20,34,28] Left hypochondrium / subcoastal region has been found to be the preferred site for Verres needle insertion.[18,28] Open Hassan technique[22] and Visiport were other alternatives used for access.[32,25] To obtain triangulation, working ports are placed away from defect or lateral to the rectus muscle.[12,13,19,20,28,34]

Whereever possible, dissection with diathermy should be avoided or used sparingly because of concerns that energy transmission can cause delayed bowel perforation.[24] Harmonic scalpel should be used with caution while doing adhesiolysis.[16,18,25,34] Hydrodissection is another alternative to divide visceral adhesions with the parietes.[11] However, sharp dissection with scissors remains the safest tool.[18,28,34,45]

A 30 degree scope would provide a good panoramic view.[13,24,28,32–35,46] Mizrahi *et al.*,[27] have used 45 degree scope for surgery.[22] In a series of 200 patients Le Blanc *et al.*,[24] managed with a 0 degree scope.[32]

Longstanding hernia with incarcerated bowel can pose technical challenge. These have a higher chance of enterotomy rate during dissection.[15] Though they are not a contraindication for laparoscopic repair.[24] Incisional hernias extending to suprapubic or xiphoid region[17] are difficult to obtain a good overlap and fixation of mesh. Another demanding situation is repair of parastomal hernia where dissection and mesh placement is around hollow viscera.[28]

In laparoscopic hernia repair mesh can be placed intraperitonealy[13,14,20,42] or in preperitoneal / extraperitoneal space.[13,18,22] At least 3 to 5 cm mesh overlap of the defect by the mesh has been recommended by different authors[13,19,20,21,24,25,27,28,33,34] to obtain good results.

Spiral tackers are placed in concentric pattern[27,32,34] along the periphery of mesh in one or two rows. The depth of penetration for the spiral tackers is < 4 mm.[47] In obese patients, the tackers may not reach up to the fascial layers. However, this should not be the reason for not offering laparoscopic incisional hernia repair to a population with raised BMI (body mass index).[17,28,32] Transfascial sutures can be helpful in such a scenario.

Transfascial sutures are used for initial orientation, to fix the mesh and to prevent mesh migration.[12,18,20,24,44,48] This technique can cause chronic postoperative pain. Sutures can be used to fix the mesh in centre or periphery. Some authors recommend transfascial sutures at the 12'o clock and the 6'o clock position.[14,31] The 3'o clock and the 9'o clock position is optional.[31]

The bowel lying underneath the mesh can be covered with omentum. This serves as a barrier. It also prevents adhesions[20] and fistulization[13,37,49] of bowel.

### Pain

In this review, 90 patients had postoperative pain related to the wound site (2.75%).

Postoperative pain can lead to readmissions, hence increasing the morbidity and costs of laparoscopic procedure.[11] This complication of laparoscopic incisional hernia repair has been found to be under reported.[15] Godney *et al.*,[50] have reported increased postoperative pain following laparoscopic ventral hernia repair when compared to open procedure. Different authors have used different period for defining chronic postoperative pain.

Sutures for mesh fixation may cause ischemic injuries to anterior abdominal wall musculature or neurovascular bundle which results in pain. Nerve entrapment in tacker is another possible explanation to the postoperative pain.[20,38] Heniford *et al.*, have reported chronic pain along the dermatomal[22] distributions. This on occasion has called for the extraction of tackers[14,28] or division of transfascial sutures.[24] Moreno-egea *et al.*,[11] found laterally placed transfixation sutures are likely to be associated with postoperative pain outside the midline. Patients tend to tolerate the mid-line transfascial fixation sutures better.[14,31] Marcaine injections, NSAIDs and narcotics have been tried to treat this complication.[22] Seromas have also been associated with chronic postoperative pain.[12] Kua *et al.*,[13] had to remove the mesh in a patient with persistent postoperative pain.

### Seromas

178 (5.45%) seromas were noted. Thirty seven of these seromas were aspirated (20.7 %). In laparoscopic hernia repair, the hernia sac is not excised.[12,15,19,22,33–35] This effectively leaves behind a potential space for seroma formation. It happens to be one of the complications inherent to this procedure.[20,22,25,51,52] Most seromas resolve with time, some requiring eight to 12 weeks for complete resolution.[15,17,19,20,22,25,27] Majority of the authors considered the seromas for conservative management. Occasionally it will give an ominous appearance of recurrence.[38,41] In such cases ultrasound of abdomen can be an useful diagnostic tool.[17,49] If followed-up using ultrasound, seromas have been reported to be ubiquitous.[53] Some surgeons[25,31,41] have advocated using dressing or abdominal binder to cause compression on abdominal wall to occlude the potential dead space. Rarely, they would require aspiration when persistent[12] or symptomatic.[16,22,24,26,33,34,54] No long-term complications have been noticed following seromas.[22] Though infection of hematoma has been noted in literature.[34]

### Recurrence

One of the most important outcome measurements of hernia repair is recurrence. On analysis, in our study we found a recurrence rate 3.67%. Some authors feel using just the tackers, without suture, increases the chances of recurrence.[14,20,33] Transfascial sutures for mesh orientation and fixation has been shown to reduce recurrence.[18,22,33] Franklin *et al.*,[20] found 73% of recurrence occurred in patients in whom transfascial sutures were not used. In these cases, only staples were relied upon to keep the mesh in place.[20] Transfascial sutures prevent the migration of prosthesis and hence recurrence.[20]

Recurrence rate is likely to improve with experience[24] and improved techniques of adequate mesh overlap at the periphery of hernia.[24] Breakdown of transfascial sutures has been attributed as one of the causes of recurrence.[24] Eid *et al.*,[19] on their long term follow-up (mean 34 months) found a recurrence rate of 5%. A rare complication is herniation of bowel between the mesh and abdominal wall.[16] Widely spaced tacks can cause bowel herniation between the tacks.[12,16] Delayed recurrence is known to occur and Le Blanc *et al.*,[25] suggest at least three-year follow-up.

### Wound infection / trocar site cellulitis

Wound infection is lower in laparoscopic hernia repair compared to open, as there is decreased extent of tissue dissection in the former.[22,55] Trocar site cellulitis seen in laparoscopic repair resolves with antibiotics.[22]

Mesh, wherever possible, should not be brought in touch with skin to avoid contamination by skin flora.[35] Leber *et al.*,[4] along with others[10,29,56–59] found polyester meshes to have highest incidence of infection, fistulisation and recurrence. Mesh infection required extraction in certain cases.[17–20,26,27] Thirty one meshes had to be extracted in 16 studies. Porcine small intestine mucosa derivative has been used in infected environment[9,60] with good results as an alternative to mesh.

### Intra-abdominal complications

Laparoscopic hernia repair causes decreased incidence of ileus.[19] This is because of less handling of the intestines and lesser tissue dissection. Ileus usually settles down spontaneously. However, mechanical obstruction should be ruled out if it tends to persist beyond 72h.[61] Ben -Haim *et al.*,[16] have reported prolonged ileus beyond five days.

Post procedure adhesive bowel obstruction increases the morbidity and may require reoperation.[13,16] Bageacu *et al.*,[14] had to perform resection anastomosis for bowel obstruction presenting more than one year after primary procedure. Obstruction was secondary to mesh complication. Dual mesh using smooth surface towards peritoneal cavity decreases the chances of adhesions. Smooth surfaced side has ePTFE, which has less postoperative visceral adhesions.[22,25,30,62–65]

Enterotomies have been reported to occur in up to 6% of patients undergoing laparoscopic ventral hernia repair.[20,23–25,43] Enterotomy during the surgery may not necessarily mean conversion to open procedure. This depends upon the expertise of the operating surgeon. Although, it does increases the rate of mesh infection.[20] Hernia repair by some surgeons was deferred in cases of recognized enterotomy.[24,31] Missed bowel perforation can have disastrous consequences. Delayed diagnosis of bowel perforation would result in mesh infection and ultimately extraction.[11,16,66] Clinical parameters need to be monitored and should be supplemented by radiological evidence if such kind of complication is suspected. Intracorporeal knotting[12,24,34] can be done to manage serosal tear of bowel, if required.

### Other complications

Postoperative fever of unknown origin prolongs the hospital stay and overall has financial bearings.[16] It has been reported in four studies.[12,16,22,27] Heniford *et al.*, performed diagnostic laparoscopy in patients with unexplained postoperative fever to exclude abdominal pathology, with negative results.[22]

In 40 (1.2 %) patients, a wound hematoma or significant bleeding at wound/trocar site was noted.[11–14,22,27,32] This is a well known complication in laparoscopic surgery. Intraabdominal pressure during laparoscopy causes tamponade effect on vessels and hence the bleeding, arterial or venous, may manifest after surgery.[67]

Enterocutaneous fistula was reported in two cases in a study by Bageacu *et al*.,[14] Both the cases required surgical intervention for treatment. There have only been two deaths reported in the review[23,44] (0.06%).

### Conversion to open

Seventy four (2.26%) cases required to be converted to open procedure. Technical difficulties in dissection and defining the right plane can result in conversion.[22] Surgeons experience is also an important factor towards conversion to open repair. Malignancy,[28] dense adhesions[16,20,24,25,28,32] and incarcerated hernia[22,35] remain common contributory factors towards conversion. Bowel injury[16,19,24,25] which cannot be dealt with laparoscopically[20] is another cause for conversion. Spillage of enteral contents increases the chances of wound infection.

## CONCLUSION

Laparoscopic repair of incisional hernia has a learning curve and increased operative time.[14,32] This form of surgery requires advanced laparoscopic skills.[16,32] It has been recommended for incisional and ventral hernia greater than 3 cm in diameter.[11,17,22,24,32,35,60,61,68]

LIVHR has shown promising results and is being widely accepted. It results in shorter hospital stay and lower short-term complications when compared to open repair.[26] Less incidence of wound infection, early return of bowel activity and faster resumption of normal activities[22,34,37,60,69] favor LIVHR. Shorter hospital stay is translated into lesser overall cost of procedure.[34] It is also feasible to perform this procedure as day case surgery.[11,25,31,32] Monro-Egea *et al.*,[11] performed 76% of cases as day care.

Laparoscopic repair allows viewing of hernia defects, which are not apparent clinically and treat multiple hernias located in different quadrants of abdomen through same incision.[13,35] It also allows dissection in the right anatomical plane.[35]

Seromas tend to be a common complication with LIVHR, which tends to settle down conservatively. Placing tacks and transfascial sutures judiciously can avoid recurrence and chronic postoperative pain. Meticulous tissue plane dissection can avoid bowel perforation. Hernias included in this study are clinically a heterogeneous group. We feel this provides strength to the study as a greater cross-section of incisional and ventral hernia was studied. The data from the observational studies has not been combined statistically but essentially summarized and this makes it a qualitative review. The evidence provided by this study would be 2++ according to grading system of “Levels of evidence”. More prospective randomized controlled trials are required to establish the role of Laparoscopic incisional and ventral hernia repair.

## References

[1] Cobb WS, Kercher KW, Heniford BT (2005). Laparoscopic repair of incisional hernias. Surg Clin North Am.

[2] De Maria EJ, Moss JM, Sugerman HJ (2000). Laparoscopic intraperitoneal polytetrafluoroethylene prosthetic patch repair of ventral hernia. Surg Endosc.

[3] Mudge M, Hughes LE (1985). Incisional hernia: A 10 year prospective study of incidence and attitudes. Br J Surg.

[4] Leber GE, Garb JL, Alexander AI, Reed WD (1998). Long term complications associated with prosthetic repair of incisional hernias. Arch Surg.

[5] Le Blanc KA, Booth WV (1993). Laparoscopic repair of incisional abdominal hernias using expanded polytetrafluoroethylene: Preliminary findings. Surg Laparosc Endosc.

[6] Schoetz DJ, Coeller JA, Veidenheimer MC (1988). Closure of abdominal wounds with polydioxanone: A prospective study. Arch Surg.

[7] Bucknall TE, Cox PJ, Ellis H (1982). Burst abdomen and incisional hernia: A prospective study of 1129 major laparotomies. Br Med J (Clin Res Ed).

[8] Hesselink VJ, Luijendik RW, de Wilt JH, Heide R, Jeekel J (1993). An evaluation of risk factors in incisional hernia recurrence. Surg Gynecol Obstet.

[9] Santora TA, Roslyn JJ (1993). Incisional hernia. Surg Clin North Am.

[10] Stoppa RE (1989). The treatment of complicated groin and incisional hernias. World J Surg.

[11] Moreno-Egea A, Castillo JA, Girela E, Canteras M, Aguayo JL (2002). Outpatient laparoscopic incisional / ventral hernioplasty: Our experience in 55 cases. Surg Lap Endosc Percut Tech.

[12] Aura T, Habib E, Mekkaoui M, Brassier D, Elhadad A (2002). Laparoscopic tension-free repair of anterior abdominal wall incisional and ventral hernias with an intraperitoneal Gore-Tex mesh: Prospective study and review of literature. J Laparoendosc Adv Surg Tech A.

[13] Kua KB, Coleman M, Martin I, O'Rourke N (2002). Laparoscopic repair of ventral incisional hernia. ANZ J Surg.

[14] Bageacu S, Blanc P, Breton C, Gonzales M, Porcheron J, Chabert M (2002). Laparoscopic repair of incisional hernia: A retrospective study of 159 patients. Surg Endosc.

[15] Bamehriz F, Birch DW (2004). The feasibility of adopting laparoscopic incisional hernia repair in general surgery practice: Early outcomes in an unselected series of patients. Surg Laparosc Endosc Percutan Tech.

[16] Ben-Haim M, Kuriansky J, Tal R, Zmora O, Mintz Y, Rosin D (2002). Pitfalls and complications with laparoscopic intraperitoneal expanded polytetrafluoroethylene patch repair of postoperative ventral hernia. Surg Endosc.

[17] Bower CE, Reade CC, Kirby LW, Roth JS (2004). Complications of laparoscopic incisional-ventral hernia repair: The experience of a single institution. Surg Endosc.

[18] Chowbey PK, Sharma A, Khullar R, Soni V, Baijal M (2003). Laparoscopic ventral hernia repair with extraperitoneal mesh: Surgical technique and early results. Surg Laparosc Endosc Percutan Tech.

[19] Eid GM, Prince JM, Mattar SG, Hamad G, Ikrammudin S, Schauer PR (2003). Medium-term follow-up confirms the safety and durability of laparoscopic ventral hernia repair with PTFE. Surgery.

[20] Franklin ME, Gonzalez JJ, Glass JL, Manjarrez A (2004). Laparoscopic ventral and incisional hernia repair: An 11-year experience. Hernia.

[21] Franklin ME, Dorman JP, Glass JL, Balli JE, Gonzalez JJ (1998). Laparoscopic ventral incisional hernia repair. Surg Laparosc Endosc.

[22] Heniford BT, Park A, Ramshaw BJ, Voeller G (2000). Laparoscopic ventral and incisional hernia repair in 407 patients. J Am Coll Surg.

[23] Koehler RH, Voeller G (1999). Recurrences in Laparoscopic Incisional hernia repairs: A personal series and review of the literature. J Soc Laparoendosc Surg.

[24] LeBlanc KA, Whitaker JM, Bellanger DE, Rhynes VK (2003). Laparoscopic incisional and ventral hernioplasty: Lessons learned from 200 patients. Hernia.

[25] LeBlanc KA, Booth WV, Whitaker JM, Bellanger DE (2000). Laparoscopic incisional and ventral herniorraphy in 100 patients. Am J Surg.

[26] McGreevy JM, Goodney PP, Birkmeyer CM, Finlayson SR, Laycock WS, Birkmeyer JD (2003). A prospective study comparing the complication rates between laparoscopic and open ventral hernia repairs. Surg Endosc.

[27] Mizrahi S, Lantsberg L, Kirshtein B, Bayme M, Avinoah E (2003). The experience with a modified technique for laparoscopic ventral hernia repair. J Laparoendosc Adv Surg Tech A.

[28] Muysoms F, Daeter E, Vander Mijnsbrugge G, Claeys D (2004). Laparoscopic intraperitoneal repair of incisional and ventral hernias. Acta Chir Belg.

[29] Park A, Birch DW, Lovrics P (1998). Laparoscopic and open incisional hernia repair: A comparison study. Surgery.

[30] Park A, Gagner M, Pomp A (1996). Laparoscopic repair of large incisional hernias. Surg Laparosc Endosc.

[31] Parker HH, Nottingham JM, Bynoe RP, Yost MJ (2002). Laparoscopic repair of large incisional hernias. Am Surg.

[32] Raftopoulos I, Vanuno D, Khorsand J, Ninos J, Kouraklis G, Lasky P (2002). Outcome of laparoscopic ventral hernia repair in correlation with obesity, type of hernia and hernia size. J Laparoendosc Adv Surg Tech A.

[33] van't Riet M, Vrijland WW, Lange JF, Hop WC, Jeekel J, Bonjer HJ (2002). Mesh repair of incisional hernia: Comparison of laparoscopic and open repair. Eur J Surg.

[34] Olmi S, Magnone S, Erba Luigi (2005). Results of Laparoscopic versus open abdominal and incisional hernia repair. J Soc Laproendosc Surg.

[35] Verbo A, Petito L, Pedretti G, Lurati M, D'Alba P, Coco C (2004). Use of a new type of PTFE mesh in Laparoscopic incisional hernia repair: The continuing evolution of technique and surgical expertise. Int Surg.

[36] Chari R, Chari V, Eisenstat M, Chung R (2000). A case controlled study of laparoscopic incisional hernia repair. Surg Endosc.

[37] Holzman MD, Purut CM, Reintgen K, Eubanks S, Pappas TN (1997). Laparoscopic ventral and incisional hernioplasty. Surg Endosc.

[38] Lau H, Patil NG, Yuen WK Lee F (2002). Laparoscopic incisional hernioplasty utilising on-lay expanded polytetrafluoroethylene DualMesh: Prospective study. Hong Kong Med J.

[39] Tagaya N, Mikami H, Aoki H, Kubota K (2004). Long term complications of laparoscopic ventral and incisional hernia repair. Surg Laparosc Endosc Percutan Tech.

[40] Tsimoyiannis EC, Tassis A, Glantzounis G, Jabarin M, Siakas P, Tzourou H (1998). Laparoscopic intraperitoneal onlay mesh repair of incisional hernia. Surg Laparosc Endosc.

[41] Misra MC, Bansal VK, Kulkarni MP, Pawar DK (2006). Comparison of laparoscopic and open repair of incisional and primary ventral hernia: Results of a prospective randomised study. Surg Endosc.

[42] Lomanto D, Iyer SG, Shabbir A, Cheah WK (2006). Laparoscopic versus open ventral hernia mesh repair: A prospective study. Surg Endosc.

[43] Earle D, Seymour N, Fellinger E, Perez A (2006). Laparoscopic versus open incisional hernia repair. Surg Endosc.

[44] Motson RW, Engledow AH, Medhurst C, Adib R, Warren SJ (2006). Laparoscopic incisional hernia repair with a self-centring suture. Br J Surg.

[45] Ramshaw BJ, Esartia P, Schwab J, Mason EM, Wilson RA, Duncan TD (1999). Comparison of Laparoscopic and open ventral herniorrhaphy. Am Surg.

[46] Rosen M, Brody F, Ponsky J, Walsh RM, Rosenblatt S, Duperier F (2003). Recurrence after laparoscopic ventral hernia repair. Surg Endosc.

[47] Carbajo MA, Martin del Olmo JC, Blanco JL, de la Cuesta C, Toledano M, Martin F (1999). Laparoscopic treatment vs. open surgery in the solution of major incisional and abdominal wall hernias with mesh. Surg Endosc.

[48] van't Riet M, De Vos van Steenwijk PJ, Kleinrensink GJ, Steyerberg EW, Bonjer HJ (2002). Tensile strength of mesh fixation methods in laparoscopic incisional hernia repair. Surg Endosc.

[49] Moreno-Egea A, Liron R, Girela E, Aguayo JL (2001). Laparoscopic repair of ventral and incisional hernias using a new composite mesh. Surg Laparosc Endosc Percutan Tech.

[50] Goodney PP, Birkmeyer CM, Birkmeyer JD (2002). Short-term outcomes of laparoscopic and open ventral hernia repair: A meta-analysis. Arch Surg.

[51] Heniford BT, Le Blanc KA, Voeller GR (2001). Laparoscopic repair of incisional hernias: Surgical technique. Contemp Surg.

[52] Chowbey PK, Sharma A, Khullar R, Mann V, Baijal M, Vashistha A (2000). Laparoscopic ventral hernia repair. J Laparoendosc Adv Surg Tech A.

[53] Susmallian S, Gewurtz G, Ezri T, Charuzi I (2001). Seroma after laparoscopic repair of hernia with PTFE patch: Is it really a complication?. Hernia.

[54] Varghese TK, Denham DW, Dawes LG, Murayama KM, Prystowsky JB, Joehl RJ (2002). Laparoscopic ventral hernia repair: An initial institutional experience. J Surg Res.

[55] Robbins SB, Pofhal WE, Gonzalez RP (2001). Laparoscopic ventral hernia repair reduces wound complications. Am Surg.

[56] Temudom T, Siadati M, Sarr MG (1996). Repair of complex giant or recurrent ventral hernias by using tension free intraperitoneal prosthetic mesh (Stoppa technique): Lessons learned from our initial experience (fifty patients). Surgery.

[57] George CD, Ellis H (1986). The results of incisional hernia repair: A twelve year review. Ann R Coll Surg Engl.

[58] Bauer JJ, Salky BA, Gelernt IM, Kreel I (1987). Repair of large abdominal wall defects with expanded polytetrafluoroethylene (PTFE). Ann Surg.

[59] Amid PK, Shulman AG, Lichtenstein IL, Sostrin S, Young J, Hakakha M (1994). Experimental evaluation of a new composite mesh with the selective property of incorporation to the abdominal wall without adhering to the intestines. J Biomed Mater Res.

[60] Franklin ME, Gonzalez JJ, Glass JL (2004). Use of porcine small intestinal submucosa as a prosthetic device for laparoscopic repair of hernias in contaminated fields: 2-year follow-up. Hernia.

[61] Cushieri A, Steele RJC, Moosa AR (2002). Disorders of the abdominal wall and peritoneal cavity. Essential Surg Pract.

[62] Law NW (1990). A comparison of polypropylene mesh, expanded polytetrafluoroethylene patch and polyglycolic acid mesh for the repair of experimental abdominal wall defects. Acta Chir Scand.

[63] Gillion JF, Begin GF, Marecos C (1997). Expanded PTFE patches used in the intraperitoneal or extraperitoneal position for repair of incisional hernias of the anterolateral abdominal wall. Am J Surg.

[64] Costanza MJ, Heniford BT, Arca MJ, Mayes JT, Gagner M (1998). Laparoscopic repair of recurrent ventral hernia. Am Surg.

[65] Toy FK, Bailey RW, Carey S, Chappuis CW, Gagner M, Josephs LG (1998). Prospective multicenter study of laparoscopic ventral hernioplasty: Preliminary results. Surg Endosc.

[66] Berger D, Bientzle M, Muller A (2002). Postoperative complications after laparoscopic incisional hernia repair: Incidence and treatment. Surg Endosc.

[67] Le Blanc KA (2004). Laparoscopic incisional and ventral hernia repair: Complications-How to avoid and handle. Hernia.

[68] Sander LM, Flint LM, Ferrara JJ (1999). Initial experience with laparoscopic repair of incisional hernias. Am J Surg.

[69] Larson GM (2000). Ventral hernia repair by the laparoscopic approach. Surg Clin North Am.

